# Cold Shock Induced Protein RBM3 but Not Mild Hypothermia Protects Human SH-SY5Y Neuroblastoma Cells From MPP^+^-Induced Neurotoxicity

**DOI:** 10.3389/fnins.2018.00298

**Published:** 2018-05-03

**Authors:** Hai-Jie Yang, Xiang Shi, Fei Ju, Bei-Ning Hao, Shuang-Ping Ma, Lei Wang, Bin-Feng Cheng, Mian Wang

**Affiliations:** ^1^School of Life Science and Technology, Xinxiang Medical University, Xinxiang, China; ^2^Henan Collaborative Innovation Center of Molecular Diagnosis and Laboratory Medicine, Xinxiang Medical University, Xinxiang, China; ^3^Linzhou No.1 Middle School, Anyang, China

**Keywords:** mild hypothermia, RBM3, MPP^+^, Parkinson's disease, neuroprotection, apoptosis

## Abstract

The cold shock protein RBM3 can mediate mild hypothermia-related protection in neurodegeneration such as Alzheimer's disease. However, it remains unclear whether RBM3 and mild hypothermia provide same protection in model of Parkinson's disease (PD), the second most common neurodegenerative disorder. In this study, human SH-SY5Y neuroblastoma cells subjected to insult by 1-methyl-4-phenylpyridinium (MPP^+^) served as an *in-vitro* model of PD. Mild hypothermia (32°C) aggravated MPP^+^-induced apoptosis, which was boosted when RBM3 was silenced by siRNA. In contrast, overexpression of RBM3 significantly reduced this apoptosis. MPP^+^ treatment downregulated the expression of RBM3 both endogenously and exogenously and suppressed its induction by mild hypothermia (32°C). In conclusion, our data suggest that cold shock protein RBM3 provides neuroprotection in a cell model of PD, suggesting that RBM3 induction may be a suitable strategy for PD therapy. However, mild hypothermia exacerbates MPP^+^-induced apoptosis even that RBM3 could be synthesized during mild hypothermia.

## Introduction

Parkinson's disease (PD) is the second most common neurodegenerative disorder worldwide (de Lau and Breteler, 2006). It is featured by a progressive loss of dopaminergic neurons in substantia nigra pars compacta (Spillantini et al., 1997). Although nigral dopaminergic cellular apoptosis and mitochondrial dysfunction induced by various factors may play a critical role in the neurodegenerative processes in PD, the etiology of PD remains elusive (Yuan and Yankner, 2000; Lin and Beal, 2006; Yang et al., 2010). Even so, researchers are searching for factors, as yet undiscovered, that could prevent dopaminergic neuronal apoptosis and serve as possible therapeutic strategies of PD (Ye et al., 2015; Zhou R. B. et al., 2017).

Mild hypothermia (32–35°C) is a well-established therapeutic tool used to alleviate neural injury from various disorders, including hypoxic-ischemic encephalopathy in newborn infants, and acute brain injuries (Edwards et al., 2010; Jacobs et al., 2013). Hypothermia can also reduce neuronal death in neurodegenerative disease, which is known to involved the progressive loss of neurons (Lin and Beal, 2006). For example, mild hypothermia has also been shown to provide neuroprotective effects in mouse model of Alzheimer's disease (Peretti et al., 2015), the most common neurodegenerative disorder, indicating a potential role of mild hypothermia in therapy for neurodegenerative diseases. However, the therapeutic effects of mild hypothermia on PD, the second most common neurodegenerative disorder, has not been investigated.

The cold shock protein RBM3 (RNA-binding motif 3) gene, encoding a 157-amino acid protein with a molecular weight of 17 kD, can be stimulated in response to mild hypothermia (Derry et al., 1995; Danno et al., 1997). Substantial evidence has shown that RBM3 acts as an important mediator of mild hypothermia in neuroprotection (Chip et al., 2011). In mouse models of degenerative diseases (Peretti et al., 2015), RBM3 was found to mediate structural plasticity and protective effects of cooling against neuron loss. *In vitro*, RBM3 confers clear neuroprotective effects from apoptosis induced by various insults, such as serum and glucose deprivation, staurosporine, H_2_O_2_, nitric oxide (NO), retinoic acid (RA), and UV irradiation (Ferry et al., 2011; Wellmann et al., 2011; Ma et al., 2017; Yang et al., 2017; Zhuang et al., 2017). As an RNA-binding protein, RBM3 may promote neural cell survival by accelerating ribosome assembly, affecting microRNA biosynthesis, stabilizing mRNA structure, and increasing global *de novo* protein synthesis (Zhu et al., 2016; Zhou R. B. et al., 2017). However, it remains unclear whether RBM3 can provide protection in PD models. The gene expression patterns of RBM3 during PD progression remains largely unknown (Zhu et al., 2016).

The apoptosis induced by 1-methyl-4-phenylpyridinium (MPP^+^) in SH-SY5Y human neuroblastoma cells is a well-known *in vitro* model of dopaminergic (DAergic) neurons for PD (Bloem et al., 1990; Falkenburger et al., 2016). Utilizing this model, we attempted to investigate whether both RBM3 and mild hypothermia prevents MPP^+^-induced apoptosis in SH-SY5Y cells. Meanwhile, we also examined the effects of MPP^+^ on RBM3 expression in SH-SY5Y cells. This work aimed to present evidences for RBM3 induction/overexpression as a potential therapeutic strategy against PD.

## Materials and methods

### Plasmid constructions and transfection

Full-length human RBM3 coding domain sequence was cloned into the vector pIRES2-EGFP/myc within the restriction sites of *Bgl* II and *Sma* I. Human SH-SY5Y neuroblastoma cells and HEK293 cells were transfected with pIRES2-EGFP/myc-RBM3 construct or with the empty vector pIRES2-EGFP/myc. Transfections were performed using Lipofectamine 2000 according to the manufacturer's instructions. In all experiments, cells were subjected to further treatment 2 d after transfection (Yang et al., 2017).

### Cell culture and treatment

Human SH-SY5Y neuroblastoma cells were cultured in Dulbecco's modified Eagle's medium (Gibco) supplemented with 10% fetal calf serum (FCS; Hyclone), 100 U/ml penicillin and 100 μg/ml streptomycin at 37°C in 5% CO_2_.

MPP^+^ iodide (Sigma) was added to the medium to a final concentration of 1, 2, or 3 mM. Prior to the experimentation, the SH-SY5Y cells were plated at a density of ~3.1 × 10^4^ cells per cm^2^ area in 96-well plates (for MTT, Caspase3/7, and TUNEL assays) and 5.5 × 10^4^ cells per cm^2^ area in 6-well plates (for Western blot and qPCR assays) and allowed to incubate for 24 h. The cells were then treated with MPP^+^.

### Quantitative PCR (qPCR)

Total RNA was extracted from the cells using Total RNA Isolation Kit (Dingguo, Beijing) and the cDNA was synthesized using an EasyScript™ Reverse Transcription Kit (ABM). Quantitative real-time PCR reactions were performed on 7500 Real Time PCR System (Thermo) using EvaGreen 2 × qPCR MasterMix (abm). Primer sequences used were as follows: hsa-rbm3 forward primer: 5′-TGG GAG GGC TCA ACT TTA ACA-3′; hsa-rbm3 reverse primer: 5′-GTC TCC CGG TCC TTG ACA AC-3′; hsa-gapdh forward primer: 5′-TGC CCT CAA CGA CCA CTT TG-3′; hsa-gapdh reverse primer: 5′-TAC TCC TTG GAG GCC ATG TG-3′.

### Western blot

After treatment, cells in 6-well plate were washed twice with cold PBS (3.2 mM Na_2_HPO_4_, 0.5 mM KH_2_PO_4_, 1.3 mM KCl, 140 mM NaCl, pH 7.4) and then lysed in ice-cold lysis buffer (20 mM Tris–HCl, pH 7.5, 150 mM NaCl, 1 mM EDTA, 0.5% Triton X-100, 2.5 mM sodium pyrophosphate, 1 mM β-glycerolphosphate, 1 mM Na_3_VO_4_, 1 mM PMSF, and Roche's complete protease cocktail inhibitors) and centrifuged at 15,000 × g for 15 min at 4°C. The proteins in the supernatant were measured using a Protein Assay Kit II (BioRad, U.S.). Then 10–50 μg of protein samples was separated by 12–15% SDS-PAGE and transferred onto a polyvinylidene difluoride membrane (Millipore, U.S.). After blocking with PBS-T (0.1% Tween-20 in PBS) containing 5% non-fat milk, the membranes were incubated with different primary antibodies: RBM3 (Sigma, 1:5000), cleaved PARP-1 (Cell Signaling Tech., 1:1000), and β-actin (Sigma, 1:5000). The membranes were further incubated with horseradish peroxidase-conjugated secondary antibodies and developed using Pierce's West Pico Chemiluminescence substrate. To determine the equivalence of amounts of loaded protein, the developed membranes were stripped and subjected to detection with anti-β-actin antibodies (Yang et al., 2008). In some cases, immunoblots were quantified by measuring the immunoreactive protein band density with the software ImageJ 1.50 (NIH, U.S.).

### Cell viability assay

MPP^+^-induced cytotoxicity *in vitro* was assessed using MTT staining as described previously (Yang et al., 2017). In brief, 1 × 10^4^ cells were seeded onto 96-well plates in a sextuplet manner. Following overnight culture, medium was supplemented with MPP^+^ at various concentrations for 24 h. The plates were stained with 10% MTT in complete medium for 4–6 h, and then the stained cells were dissolved with 100 μl DMSO. After 10 min of incubation, the dye extracts were measured at a wavelength of 490 nm with Tecan SpectraMaxPlus microplate reader (Molecular Devices, U.S.). Absolute reading values were normalized by scaling to the mean of untreated SH-SY5Y or pIRES2-EGFP/myc-transfected SH-SY5Y cell culture grown in complete medium alone (defined as 1). These experiments were performed in triplicate.

### Caspase3/7 luciferase assay

The activity of caspase 3/7 released from drug-insulted SH-SY5Y cells was determined using Caspase-Glo®3/7 Assay (Promega, U.S.) according to the manufacturer's instructions. Briefly, 1 × 10^4^ cells per well were seeded in 96-well plates. After 24 h of culture, the cells were treated with 0, 1, 2, and 3 mM MPP^+^ in 100 μl complete medium. After another 24 h, 100 μl caspase-Glo®3/7 reagent was added to the wells. After shaking at 300–500 rpm for 30 s, the plates were incubated for 1 h at room temperature. Finally, the activities of caspase 3/7 in cells treated with MPP^+^ were examined in triplicate using a Luminometer (Promega, U.S.).

### Short interfering RNA

To silence human RBM3 mRNA (GenBank NM_006743.4), an RBM3-specific short interfering RNA (siRNA) target the coding region was designed (Yang et al., 2017). The specific siRNA (5′ -CCA UGA ACG GAG AGU CUC UTT-3′) and a scrambled siRNA (5′ -UUC UCC GAA CGU GUC ACG UTT-3′) not matching with any of the human genes were synthesized by GenePharma (Shanghai, China). When cells in six-well plates reached to 70–80% confluence, cells were transfected with siRNA (10 nM) using Lipofectamine 2000. At 48 h post transfection, cells were used for further experiments.

### Terminal transferase dUTP nick end labeling (TUNEL) assay

Cells exhibiting DNA fragmentation were identified by TUNEL assay using a modified version of the manufacturer's recommended procedure (Roche, CA, U.S.). Typically, after 24 h of exposure to 3 mM MPP^+^, the cells were rinsed with PBS, fixed in cold 4% paraformaldehyde for 15 min, and then permeabilized with 0.2% Triton X-100 in PBS. Cells were incubated with TUNEL labeling mixture diluted (1:3) with dilution buffer in a humidified chamber at 37°C for 1 h, followed by incubation with streptavidin-peroxidase enzyme conjugates at 37°C for 30 min, and the signal was amplified with TSA (PerkinElmer) at a dilution of 1:200 for 30 min. Cells were then incubated with 4,6 diamidine-2-phenyllindole dihydrochloride (DAPI) (Sigma, U.S.) dye (5 μg/ml) for 30 min. Cells were visualized using fluorescent light microscope (Carl Zeiss, U.S.).

### Statistical analysis

The data are here expressed as mean ± S.D. of at least three independent experiments. Statistical analysis was performed using two-way ANOVA (for **Figures 3**,**4**) or one-way ANOVA (to compare two groups for other Figures). *P* values of < 0.05 were considered statistically significant.

## Results

### MPP^+^ treatment downregulates RBM3 expression at both the mRNA and protein levels

**Figure 1:**
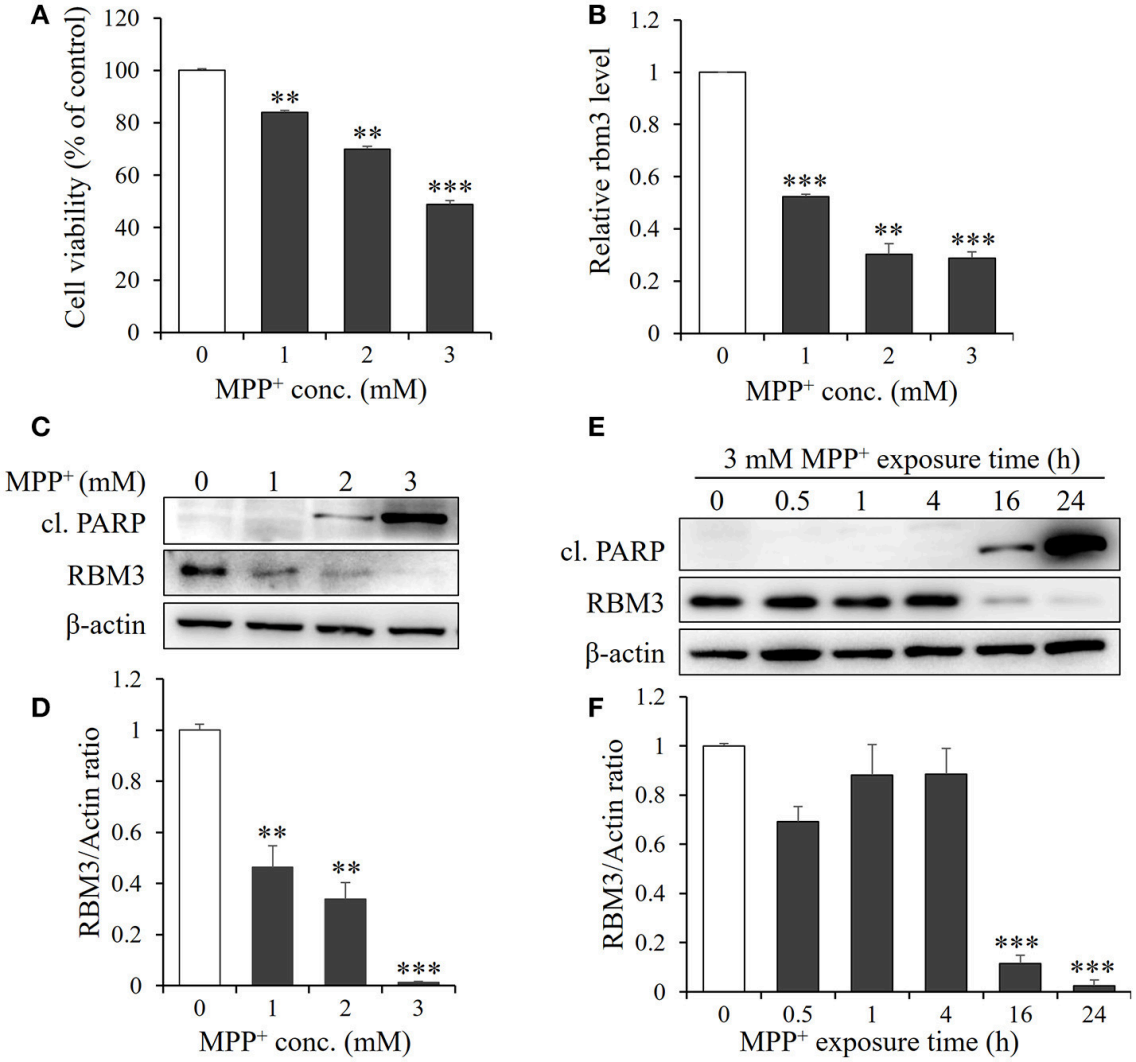
MPP^+^ treatment downregulates RBM3 expression at both mRNA and protein levels. SH-SY5H cells were treated with MPP^+^ at various concentrations (0, 1, 2, and 3 mM) for 1 d. **(A)** Cell viability was assessed by MTT assay. **(B)** The *Rbm3* mRNA expression was examined by qPCR. **(C)** The protein levels of RBM3 and cleaved (cl.) PARP were measured by Western blot. **(D)** A bar graph showed the relative levels of RBM3 in **(C). (E)** Cells were treated with 3 mM MPP^+^ for a time course as indicated, and the protein levels of RBM3 and cl. PARP were measured. **(F)** A bar graph showed the relative levels of RBM3 in **(E)**. All the data above represent the mean ± S.D. (*n* = 3). Multiple comparisons were analyzed by a one-way ANOVA and Newman-Keuls *post hoc*. ^*^Indicates *post hoc* significant difference compared to control group (37°C in the absence of MPP^+^). ^**^P < 0.01 and ^***^P < 0.001.

Exposure of SH-SY5Y cells to various concentrations of MPP^+^ for 1 d induced significant apoptosis in a dose-dependent manner (Figure 1A). Consistent with this, cleaved caspase substrate poly (ADP-ribose) polymerase (PARP), the hallmark of apoptosis, was markedly increased in response to MPP^+^ stimulation (Figures 1C,D).

Next, we examined the mRNA expression patterns of RBM3 in response to MPP^+^ treatment. The gene expression of RBM3 at transcriptional level is sensitive to MPP^+^ treatment. 1, 2, and 3 mM MPP^+^ post 24 h exposure reduced mRNA expression of RBM3 by 47.7, 69.7, and 71.3% compared to control (Figure 1B).

The protein expression patterns of RBM3 in this scenario were also analyzed. As shown in Figures 1C,D. RBM3 protein is moderately expressed at the basal level. However, with the increase in the concentration of MPP^+^, RBM3 protein was gradually downregulated and the reduction was most pronounced at 3 mM MPP^+^ exposure. Notably, the initiation of apoptosis, as evidenced by the cleavage of PARP, was accompanied by the commencement of RBM3 protein reduction (Figures 1E,F). These data suggest that MPP^+^ treatment could downregulate RBM3 expression at both the mRNA and protein levels.

### MPP^+^ inhibits induction of RBM3 by mild hypothermia in SH-SY5Y cells

**Figure 2:**
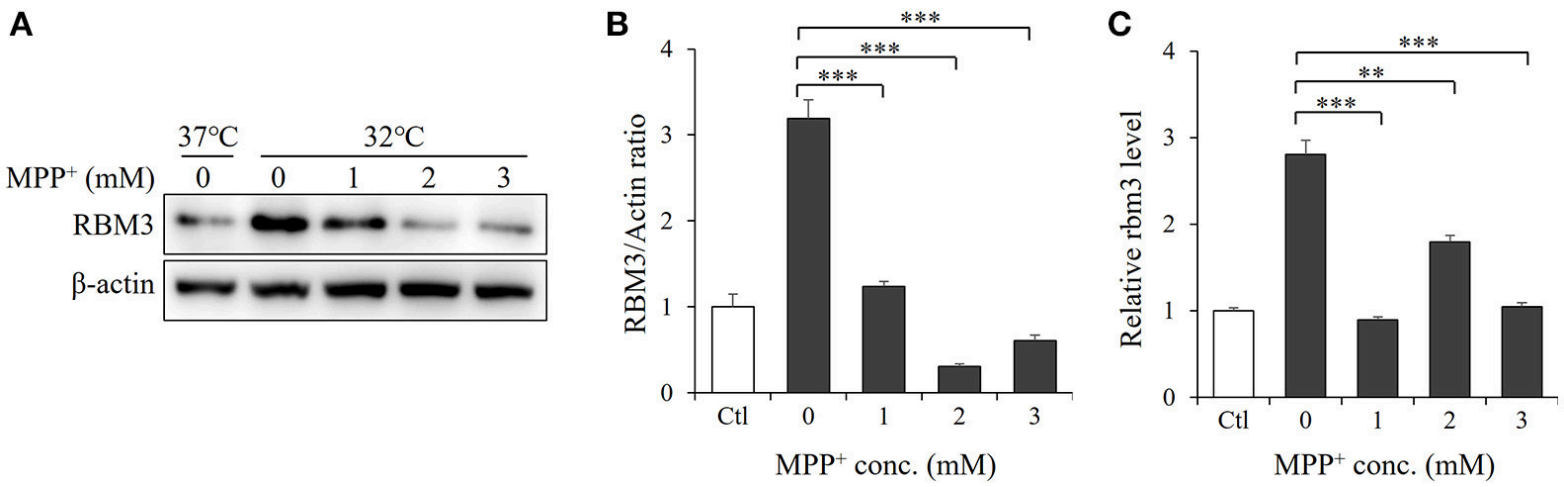
MPP^+^ inhibits RBM3 induction by mild hypothermia in SH-SY5Y cells. With the addition of MPP^+^, cells were incubated at 32°C for 1 d. **(A)** The RBM3 protein level was measured by Western blot. **(B)** A bar graph showed the relative levels of RBM3 protein in **(A)**. **(C)** The *Rbm3* mRNA expression was examined by qPCR. All the data above represent the mean ± S.D. (*n* = 3). Multiple comparisons were analyzed by a one-way ANOVA and Newman-Keuls *post hoc*. ^*^Indicates *post hoc* significant difference compared to untreated control group (32°C in the absence of MPP^+^). ^**^P < 0.01 and ^***^P < 0.001.

RBM3 is one of several products induced by mild hypothermia (Sano et al., 2015; Yang et al., 2017). As expected, SH-SY5Y cells produce it in significant quantities in response to cooling (Figures 2A,B). We next examined the effect of MPP^+^ on hypothermia-induced RBM3 expression. At the mRNA level, MPP^+^ treatment at various concentrations significantly reduced the induction of RBM3 by mild hypothermia (Figure 2C). Consistently, at the protein level, MPP^+^ treatment substantially decreased RBM3 induction under the same conditions (Figure 2A). These results indicate that MPP^+^ inhibits mild hypothermia-induced RBM3 production in SH-SY5Y cells.

### Hypothermia treatment aggravates MPP^+^-induced apoptosis in SH-SY5Y cells

**Figure 3:**
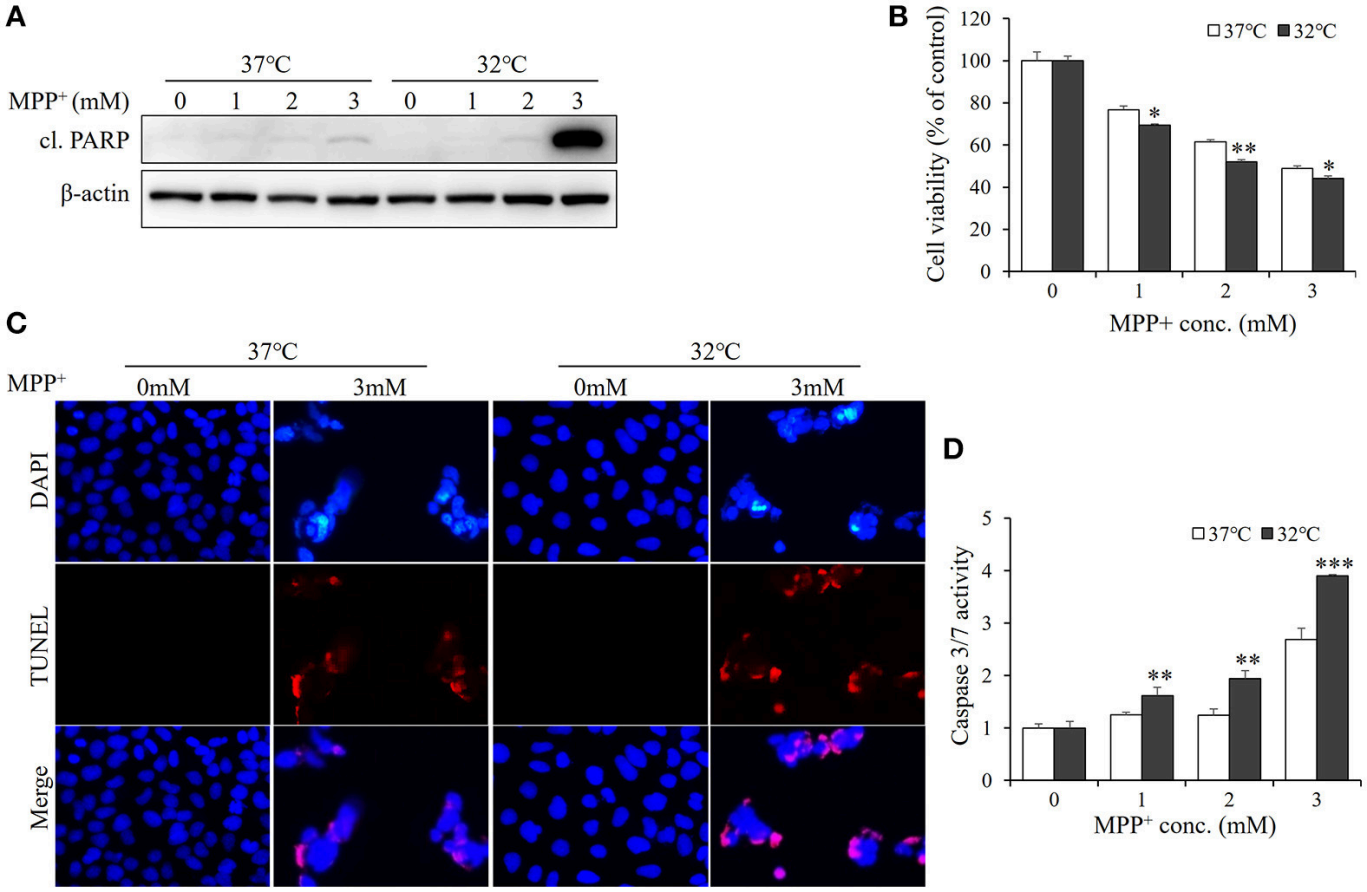
Hypothermia treatment aggravates MPP^+^-induced apoptosis in SH-SY5Y cells. Cells were pretreated with hypothermia (32°C) or normothermia (37°C) for 1 d, and then treated with various concentrations of MPP^+^ for 1 d. **(A)** Western blot was performed to detect the level of cl. PARP. **(B)** Cell viability was assessed by MTT assay. **(C)** The apoptosis was analyzed by TUNEL to figure out the DNA fragmentation caused by MPP^+^ (3 mM). The blue shows the cell nucleus, and the red exhibits DNA fragmentation. **(D)** The damage was measured by Caspase-Glo®3/7 Assay to figure out the effect of hypothermia (32°C) on MPP^+^-induced apoptosis. All the data above represent the mean ± S.D. (*n* = 3). Multiple comparisons were analyzed by a two-way ANOVA and Bonferroni's *post hoc*. ^*^Indicates *post hoc* significant difference compared to 37°C normothermia. ^*^P < 0.05, ^**^P < 0.01, and ^***^P < 0.001.

Since therapeutic cooling is widely used to rescue neural apoptosis (Antonic et al., 2014), we sought to determine whether mild hyperthermia can reduce MPP^+^-induced neurotoxicity. SH-SY5Y cells were pretreated with hypothermia (32°C) for 1 d and subjected to MPP^+^ stimulation. Unexpectedly, the levels of cleaved PARP were markedly higher in hypothermia-pretreated cells than in normothermia-treated control cells (Figure 3A, Figure S1). In line with this, the MTT assays also revealed the negative effects of mild hypothermia on the neurotoxicity of MPP^+^ (Figure 3B). Moreover, TUNEL staining showed that hypothermia pretreatment boosted MPP^+^-induced apoptosis in SH-SY5Y cells (Figure 3C), further suggesting that mild hypothermia may aggravate the apoptosis elicited by MPP^+^.

Considering that PARP is a marker of late-stage apoptosis, hallmarks of early state apoptosis, caspase-3, and−7 (Chip et al., 2011) were also examined with caspase-3/7 activation luciferase assay (Scabini et al., 2011). As shown in Figure 3D, hypothermia-pretreated cells exhibited a higher activity of caspase3/7 than normothermia-treated cells in response to MPP^+^ stimulation. Together, these data indicate that hypothermia treatment may aggravate MPP^+^-induced apoptosis in SH-SY5Y cells.

To test the effect of RBM3 induction by hypothermia on MPP^+^-induced apoptosis, RBM3 was silenced with RBM3-specific siRNA. As shown in Figure S2A, RBM3 was markedly downregulated by hypothermia. Compared to scrambled-transfected cells, RBM3-silencing cells exhibited a boosted apoptosis under hypothermic conditions, as suggested by Western blot analysis and MTT assay (Figure S2B,C).

### RBM3 overexpression rescues SH-SY5Y cells from MPP^+^-induced apoptosis

**Figure 4:**
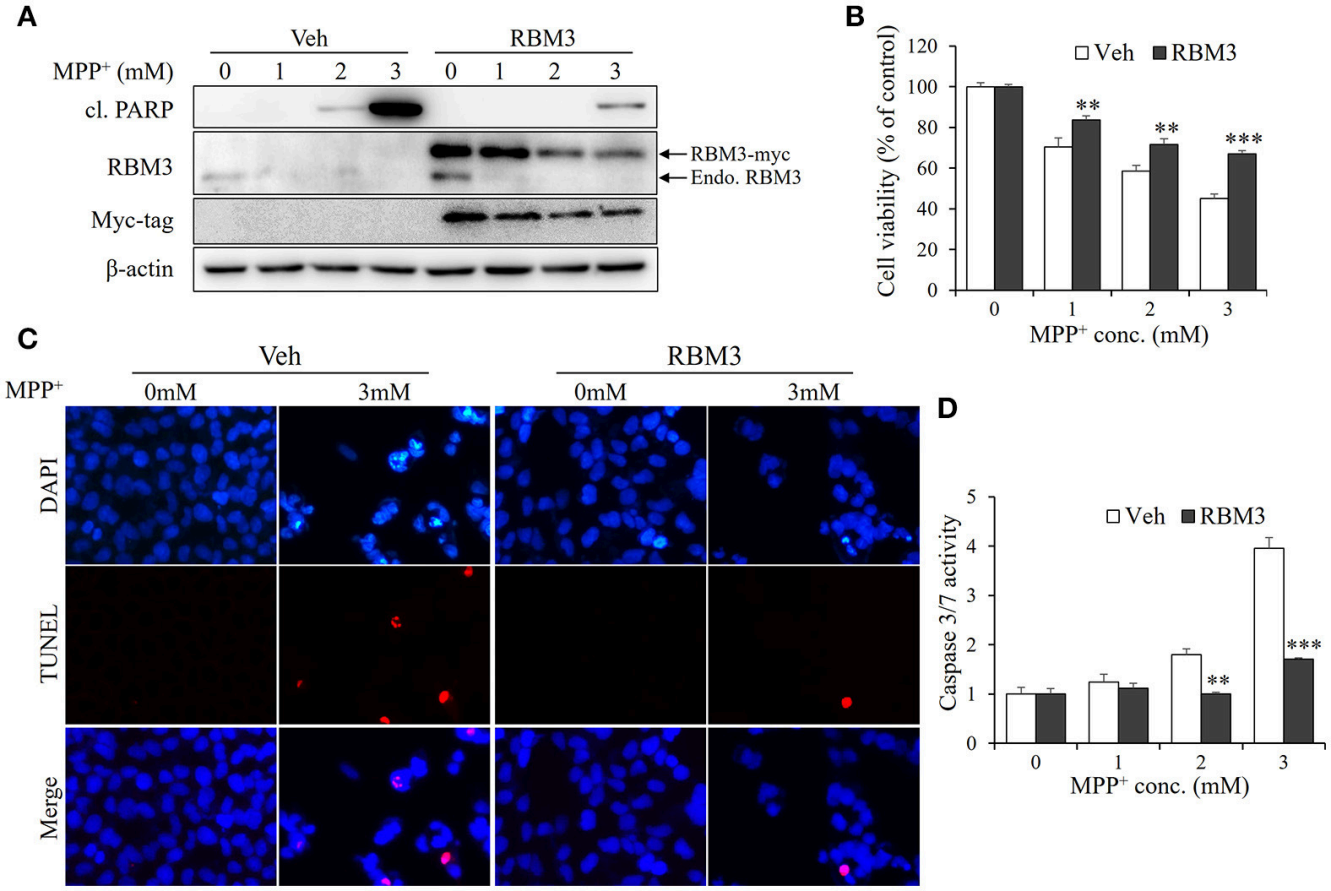
RBM3 overexpression rescues SH-SY5Y cells from MPP^+^-induced apoptosis. The RBM3-overexpressing or empty plasmid (Veh) was transfected into cells for 2 d, and treated with MPP^+^ (0, 1, 2, and 3 mM) for 1 d. **(A)** Western blot was performed to detect the protein levels of myc tag, RBM3, and cl. PARP. The upper arrow indicates myc-tagged RBM3, and the lower one indicates endogenous (endo.) RBM3. **(B)** Cell viability was assessed by MTT assay. **(C)** The apoptosis was analyzed by TUNEL to figure out the DNA fragmentation caused by MPP^+^ (3 mM). The blue shows the cell nucleus, and the red exhibits DNA fragmentation. **(D)** The damage was measured by Caspase-Glo®3/7 Assay to show the effect of RBM3 overexpression on MPP^+^-induced apoptosis. All the data above represent the mean ± S.D. (*n* = 3). Multiple comparisons were analyzed by a two-way ANOVA and Bonferroni's *post hoc*. ^*^Indicates post hoc significant difference compared to control groups (Veh). ^**^P < 0.01 and ^***^P < 0.001.

As demonstrated above, mild hypothermia could not prevent neuroblastoma cells SH-SY5Y from undergoing MPP^+^-induced apoptosis. However, RBM3 is known to be a crucial mediator in hypothermia-related neuroprotection (Chip et al., 2011; Peretti et al., 2015). For this reason, we wondered whether RBM3 could reduce the neurotoxicity associated with MPP^+^ in SH-SY5Y cells. Thus, we overexpressed myc-tagged RBM3 in SH-SY5Y cells (Figure 4A). In the presence of MPP^+^, the levels of cleaved PARP in RBM3-overexpressing cells were markedly lower than in vehicle-transfected control cells. Surprisingly, MPP^+^ not only downregulated the expression of endogenous RBM3 but also reduced the protein levels of ectopic RBM3 (Figure 4A). However, RBM3 overexpression can reduce the neurotoxicity of MPP^+^ on SH-SY5Y cells, as indicated by MTT assays (Figure 4B). This was corroborated by TUNEL staining tests, which demonstrated that RBM3 overexpression prevents MPP^+^-induced apoptosis in SH-SY5Y cells to a significant extent (Figure 4C). RBM3-overexpressing cells exhibited less caspase3/7 activity in response to MPP^+^ stimulation than control cells (Figure 4D). In summary, RBM3 could prevent MPP^+^-induced apoptosis in SH-SY5Y cells, although its protein levels declined in response to MPP^+^.

## Discussion

In this study, we demonstrated that cold shock protein RBM3 protects neuroblastoma cells SH-SY5Y from MPP^+^-induced apoptosis. Surprisingly, mild hypothermia does not possess neuroprotective effects although RBM3 is potently induced under hypothermic conditions. Our data suggest that RBM3 induction or overexpression is a potential strategy for therapy of neurodegenerative diseases such as PD.

Since the 1950s (Wang et al., 2006), therapeutic hypothermia (especially mild hypothermia) has been exploited in clinical settings to mitigate neurological injuries. Data accumulated from *in vivo* studies confirmed the neuroprotective effects of mild hypothermia in acute neurological injuries (Batchelor et al., 2013; Martirosyan et al., 2017). Currently, mild hyperthermia (33°C) is still frequently used for treatment of neonatal asphyxia (Jacobs et al., 2013). It has also been shown to improve the neurological outcome with fewer and less severe side effects in neurodegenerative disease models, including prion-infected and Alzheimer-type models (Peretti et al., 2015). For this reason, we speculated that mild hyperthermia may provide similar neuroprotection on PD cell model. Unfortunately, mild hyperthermia did not prevent MPP^+^-induced apoptosis in neuroblastoma cells SH-SY5Y. Instead, it boosted the neurotoxicity of MPP^+^. These data may indicate that mild hyperthermia either is not suitable for PD therapy or that it may only aggravate the apoptosis in MPP^+^-based PD cell model. In addition to MPP^+^ (or MPTP), rotenone, paraquat, and 6-hydroxydopamine (6-OHDA) are also used as toxins to mimic dopaminergic (DA) neuronal degeneration of PD (Falkenburger et al., 2016); for these reasons it is worthwhile to determine whether mild hyperthermia can confer neuroprotection in other PD cell models.

Although the neuroprotective mechanism of mild hypothermia is not well understood, cold shock protein RBM3 has been postulated to be an important factor in mediating the neuroprotective effects of mild hyperthermia (Chip et al., 2011; Zhou T. et al., 2017). In this study, we showed that RBM3 significantly reduces the neurotoxicity of MPP^+^ in SH-SY5Y cells by increasing cell survival rates and reducing cell apoptosis when RBM3 was overexpressed. Regarding the pro-survival role of RBM3 (Chip et al., 2011), the data are consistent with our previous observation that RBM3 confers neuroprotective effects against various insults (Ma et al., 2017; Yang et al., 2017; Zhuang et al., 2017). Meanwhile, mild hyperthermia also prevents apoptosis induced by these insults under same conditions. For this reason, it is not clear why mild hyperthermia aggravating MPP^+^-induced apoptosis occurs even when pro-survival RBM3 is potently induced. Accumulated data suggest that hypothermia preconditioning has multiple effects, such as metabolic inhibition, activation of extra- and intracellular defense mechanisms (Badjatia, 2016; Renga et al., 2017). Thus, one possible reason is that hyperthermia may also induce production of other pro-apoptotic factors, which compromise the neuroprotective effect of RBM3. Overall, hypothermia appears to be unfavorable for neuroprotection against MPP^+^.

The RNA-binding protein RBM3 is highly important in mediating the neuroprotective effects of hyperthermia, but little is known about the mechanism regulating RBM3 gene expression (Zhu et al., 2016). Our previous studies showed that RBM3 expression could not be altered in response to various stimuli (Ma et al., 2017; Yang et al., 2017; Zhuang et al., 2017). However, the present study showed that RBM3 expression is markedly downregulated by MPP^+^ at both the mRNA and protein levels. MPP^+^ stimulation also reduced the protein levels of forced RBM3 expression, which is driven by CMV promoter in a mammalian expression vector. Together, these data may reveal that MPP^+^ regulates RBM3 expression at the levels of transcription and post-translation (decay). In contrast, spinal cord injury (SCI) in mouse and rat also was found to increase RBM3 expression in neurons and astrocytes, although the duration of RBM3 expression is short (Zhang et al., 2013; Cui et al., 2014; Zhao et al., 2014). Therefore, it is worthwhile to ascertain why MPP^+^ behaves so distinctly in response to other stimuli (NO, RA, and UV) in controlling RBM3 expression.

Another question has also been raised about the role of RBM3 in neurodegenerative diseases. Specifically, it is possible that the loss of RBM3 function leads to or aggravates neurodegenerative diseases. As recently reported (Peretti et al., 2015), the gene silencing of RBM3 in prion and Alzheimer-type mice can result in synapse loss and eventually neuronal apoptosis. However, inducing endogenous RBM3 expression by cooling or by virus-mediated overexpression confers robust protection to neurons. Notably, our data indicate that MPP^+^-induced apoptosis is accompanied by the reduction of endogenous or forced RBM3 expression. Conversely, the overexpression of RBM3 reduces MPP^+^-induced apoptosis in SH-SY5Y neuroblastoma cells. In this way, it is still unclear whether the loss of RBM3 function is the cause or result of apoptosis. In the future, a MPTP-based PD mouse model should be used to determine the actual role of RBM3 in neurodegenerative diseases.

In conclusion, we have demonstrated that RBM3 confers neuroprotective effects in MPP^+^-induced apoptosis in human neuroblastoma cells. Mild hyperthermia may not be suitable for PD therapy, at least for MPTP-elicited PD. RBM3 rather than mild hyperthermia prevents MPP^+^-apoptosis in neuroblastoma cells, thereby suggesting that RBM3-specific induction/overexpression might be used as a strategy for the treatment of neurodegenerative diseases.

## Author contributions

All the authors provided important intellectual input, reviewed the content, and approved the final version of the manuscript. H-JY, XS, and MW contributed significantly, conceived and designed the experiments, analyzed the data, read, wrote, and approved the manuscript; H-JY, XS, FJ, and B-NH performed the experiments; S-PM, LW, and B-FC contributed reagents, materials, analysis tools. All the authors are agreed to be accountable for all aspects of the work in ensuring that questions related to the accuracy or integrity of any part of the work are appropriately investigated and resolved.

### Conflict of interest statement

The authors declare that the research was conducted in the absence of any commercial or financial relationships that could be construed as a potential conflict of interest.

## Supplementary material

Figure S1 Hypothermia treatment aggravates MPP^+^-induced apoptosis in SH-SY5Y cells. With the addition of MPP^+^ (3 mM), cells were incubated at 37 or 32°C for a time course as indicated, and the protein levels of cl. PARP were measured by Western blot.

Figure S2 RBM3 silencing aggravates MPP^+^-induced apoptosis in SH-SY5Y cells. After 2-d transfection of RBM3 siRNA (siRNA) or scrambled siRNA (Src), SH-SY5Y cells were pre-cultured for 1 d at 37 or 32°C and then treated with MPP^+^ (3 mM) for 1 d. Western blot was performed to detect the protein levels of RBM3 **(A)** and cl. PARP **(B)**. Under same conditions, cell viability was assessed by MTT assay **(C)**. Multiple comparisons were analyzed by a two-way ANOVA and Bonferroni's *post hoc*. ^*^Indicates *post hoc* significant difference compared to control groups (Scr). ^**^*P* < 0.01.

## References

[B1] Antonic A. Dottori M. Leung J. (2014). Hypothermia protects human neurons. Int. J. Stroke 9, 544–552. 10.1111/ijs.12224 24393199 PMC4235397

[B2] Badjatia N. (2016). Therapeutic hypothermia protocols. Handb. Clin. Neurol. 141, 619–632. 10.1016/B978-0-444-63599-0.00033-8 28190438

[B3] Batchelor P. E. Skeers P. Antonic A. Wills T. E. Howells D. W. Macleod M. R.. (2013). Systematic review and meta-analysis of therapeutic hypothermia in animal models of spinal cord injury. PLoS ONE 8:e71317. 10.1371/journal.pone.0071317 23951131 PMC3739756

[B4] Bloem B. R. Irwin I. Buruma O. J. Haan J. Roos R. A. Tetrud J. W.. (1990). The MPTP model: versatile contributions to the treatment of idiopathic Parkinson's disease. J. Neurol. Sci. 97, 273–293. 2205710 10.1016/0022-510x(90)90225-c

[B5] Chip S. Zelmer A. Ogunshola O. O. Felderhoff-Mueser U. Nitsch C. Bührer C.. (2011). The RNA-binding protein RBM3 is involved in hypothermia induced neuroprotection. Neurobiol. Dis. 43, 388–396. 10.1016/j.nbd.2011.04.010 21527344

[B6] Cui Z. Zhang J. Bao G. Xu G. Sun Y. Wang L.. (2014). Spatiotemporal profile and essential role of RBM3 expression after spinal cord injury in adult rats. J. Mol. Neurosci. 54, 252–263. 10.1007/s12031-014-0282-y 24668366

[B7] Danno S. Nishiyama H. Higashitsuji H. Yokoi H. Xue J. H. Itoh K.. (1997). Increased transcript level of RBM3, a member of the glycine-rich RNA-binding protein family, in human cells in response to cold stress. Biochem. Biophys. Res. Commun. 236, 804–807. 10.1006/bbrc.1997.7059 9245737

[B8] de Lau L. M. Breteler M. M. (2006). Epidemiology of Parkinson's disease. Lancet Neurol. 5, 525–535. 10.1016/S1474-4422(06)70471-9 16713924

[B9] Derry J. M. Kerns J. A. Francke U. (1995). RBM3, a novel human gene in Xp11.23 with a putative RNA-binding domain. Hum. Mol. Genet. 4, 2307–2311. 10.1093/hmg/4.12.2307 8634703

[B10] Edwards A. D. Brocklehurst P. Gunn A. J. Halliday H. Juszczak E. Levene M.. (2010). Neurological outcomes at 18 months of age after moderate hypothermia for perinatal hypoxic ischaemic encephalopathy: synthesis and meta-analysis of trial data. BMJ 340:c363. 10.1136/bmj.c363 20144981 PMC2819259

[B11] Falkenburger B. H. Saridaki T. Dinter E. (2016). Cellular models for Parkinson's disease. J. Neurochem. 139, 121–130. 10.1111/jnc.13618 27091001

[B12] Ferry A. L. Vanderklish P. W. Dupont-Versteegden E. E. (2011). Enhanced survival of skeletal muscle myoblasts in response to overexpression of cold shock protein RBM3. Am. J. Physiol. Cell Physiol. 301, 392–402. 10.1152/ajpcell.00098.2011 21593448 PMC3154549

[B13] Jacobs S. E. Berg M. Hunt R. Tarnow-Mordi W. O. Inder T. E. Davis P. G. (2013). Cooling for newborns with hypoxic ischaemic encephalopathy. Cochrane Database Syst. Rev. 31:CD003311 10.1002/14651858.CD003311.pub3 14583966

[B14] Lin M. T. Beal M. F. (2006). Mitochondrial dysfunction and oxidative stress in neurodegenerative diseases. Nature 443, 787–795. 10.1038/nature05292 17051205

[B15] Ma S. P. Ju F. Zhang Y. P. Shi X. Zhuang R. J. Xue H. (2017). Cold-inducible protein RBM3 protects neuroblastoma cells from retinoic acid-induced apoptosis via AMPK, p38 and JNK signaling. J. Funct. Foods 35, 175–184. 10.1016/j.jff.2017.05.045

[B16] Martirosyan N. L. Patel A. A. Carotenuto A. Kalani M. Y. Bohl M. A. Preul M. C.. (2017). The role of therapeutic hypothermia in the management of acute spinal cord injury. Clin. Neurol. Neurosurg. 154, 79–88. 10.1016/j.clineuro.2017.01.002 28131967

[B17] Peretti D. Bastide A. Radford H. Verity N. Molloy C. Martin M. G.. (2015). RBM3 mediates structural plasticity and protective effects of cooling in neurodegeneration. Nature 518, 236–239. 10.1038/nature14142 25607368 PMC4338605

[B18] Renga V. Hickey W. F. Bernat J. L. (2017). Spontaneous periodic hypothermia in Parkinson disease with hypothalamic involvement. Neurol. Clin. Pract. 7, 538–540. 10.1212/CPJ.0000000000000402 29431161 PMC5800706

[B19] Sano Y. Shiina T. Naitou K. Nakamori H. Shimizu Y. (2015). Hibernation-specific alternative splicing of mRNA encoding cold-inducible RNA-binding protein in the hearts of hamsters. Biochem. Biophys. Res. Commun. 462, 322–325. 10.1016/j.bbrc.2015.04.135 25960293

[B20] Scabini M. Stellari F. Cappella P. Rizzitano S. Texido G. Pesenti E. (2011). *In vivo* imaging of early stage apoptosis by measuring real-time caspase-3/7 activation. Apoptosis 16, 198–207. 10.1007/s10495-010-0553-1 21082356

[B21] Spillantini M. G. Schmidt M. L. Lee V. M. Trojanowski J. Q. Jakes R. Goedert M. (1997). Alpha-synuclein in lewy bodies. Nature 388, 839–840. 10.1038/42166 9278044

[B22] Wang H. Olivero W. Wang D. Lanzino G. (2006). Cold as a therapeutic agent. Acta. Neurochir. 148, 565–570. 10.1007/s00701-006-0747-z 16489500

[B23] Wellmann S. Truss M. Bruder E. Tornillo L. Eelmer A. Seeger K.. (2011). The RNA-binding protein RBM3 is required for cell proliferation and protects against serum deprivation-induced cell death. Pediatr. Res. 67, 35–41. 10.1203/PDR.0b013e3181c13326 19770690

[B24] Yang H. J. Ju F. Guo X. X. Ma S. P. Wang L. Cheng B. F.. (2017). RNA-binding protein RBM3 prevents NO-induced apoptosis in human neuroblastoma cells by modulating p38 signaling and miR-143. Sci. Rep. 7:41738. 10.1038/srep41738 28134320 PMC5278414

[B25] Yang H. J. Wang L. Xia Y. Y. Chang P. N. Feng Z. W. (2010). NF-kappaB mediates MPP^+^-induced apoptotic cell death in neuroblastoma cells SH-EP1 through JNK and c-Jun/AP-1. Neurochem. Int. 56, 128–134. 10.1016/j.neuint.2009.09.010 19778565

[B26] Yang H. Xia Y. Lu S. Q. Soong T. W. Feng Z. W. (2008). Basic fibroblast growth factor-induced neuronal differentiation of mouse bone marrow stromal cells requires FGFR-1, MAPK/ERK, and transcription factor AP-1. J. Biol. Chem. 283, 5287–5295. 10.1074/jbc.M706917200 18171671

[B27] Ye X. Han Y. Zhang L. Liu W. Zuo J. (2015). MTERF4 regulates the mitochondrial dysfunction induced by MPP^+^ in SH-SY5Y cells. Biochem. Biophys. Res. Commun. 464, 214–220. 10.1016/j.bbrc.2015.06.119 26102036

[B28] Yuan J. Yankner B. A. (2000). Apoptosis in the nervous system. Nature 407, 802–809. 10.1038/35037739 11048732

[B29] Zhang J. Li D. Shen A. Mao H. Jin H. Huang W.. (2013). Expression of RBMX after spinal cord injury in rats. J. Mol. Neurosci. 49, 417–429. 10.1007/s12031-012-9914-2 23180094

[B30] Zhao W. Xu D. Cai G. Zhu X. Qian M. Liu W.. (2014). Spatiotemporal pattern of RNA-binding motif protein 3 expression after spinal cord injury in rats. Cell. Mol. Neurobiol. 34, 491–499. 10.1007/s10571-014-0033-1 24570111 PMC11488955

[B31] Zhou R. B. Lu X. L. Zhang C. Y. Yin D. C. (2017). RNA binding motif protein 3: a potential biomarker in cancer and therapeutic target in neuroprotection. Oncotarget 8, 22235–22250. 10.18632/oncotarget.14755 28118608 PMC5400660

[B32] Zhou T. Liang Y. Jiang L. Yu T. Zeng C. Tao E. (2017). Mild hypothermia protects against oxygen glucose deprivation/reoxygenation-induced apoptosis via the Wnt/β-catenin signaling pathway in hippocampal neurons. Biochem. Biophys. Res. Commun. 486, 1005–1013. 10.1016/j.bbrc.2017.03.153 28365156

[B33] Zhu X. Bührer C. Wellmann S. (2016). Cold-inducible proteins CIRP and RBM3, a unique couple with activities far beyond the cold. Cell. Mol. Life Sci. 73, 3839–3859. 10.1007/s00018-016-2253-7 27147467 PMC5021741

[B34] Zhuang R. J. Ma J. Shi X. Ju F. Ma S. P. Wang L.. (2017). Cold-inducible protein RBM3 protects UV irradiation-induced apoptosis in neuroblastoma cells by affecting p38 and JNK pathways and Bcl2 family proteins. J. Mol. Neurosci. 63, 142–151. 10.1007/s12031-017-0964-3 28831692

