# Supplementary figures and images for: Cold Shock Induced Protein RBM3 but Not Mild Hypothermia Protects Human SH-SY5Y Neuroblastoma Cells From MPP+-Induced Neurotoxicity
Source: Front Neurosci. 2018 May 3;12:298. doi: 10.3389/fnins.2018.00298 (PMC5943555; doi:10.3389/fnins.2018.00298)

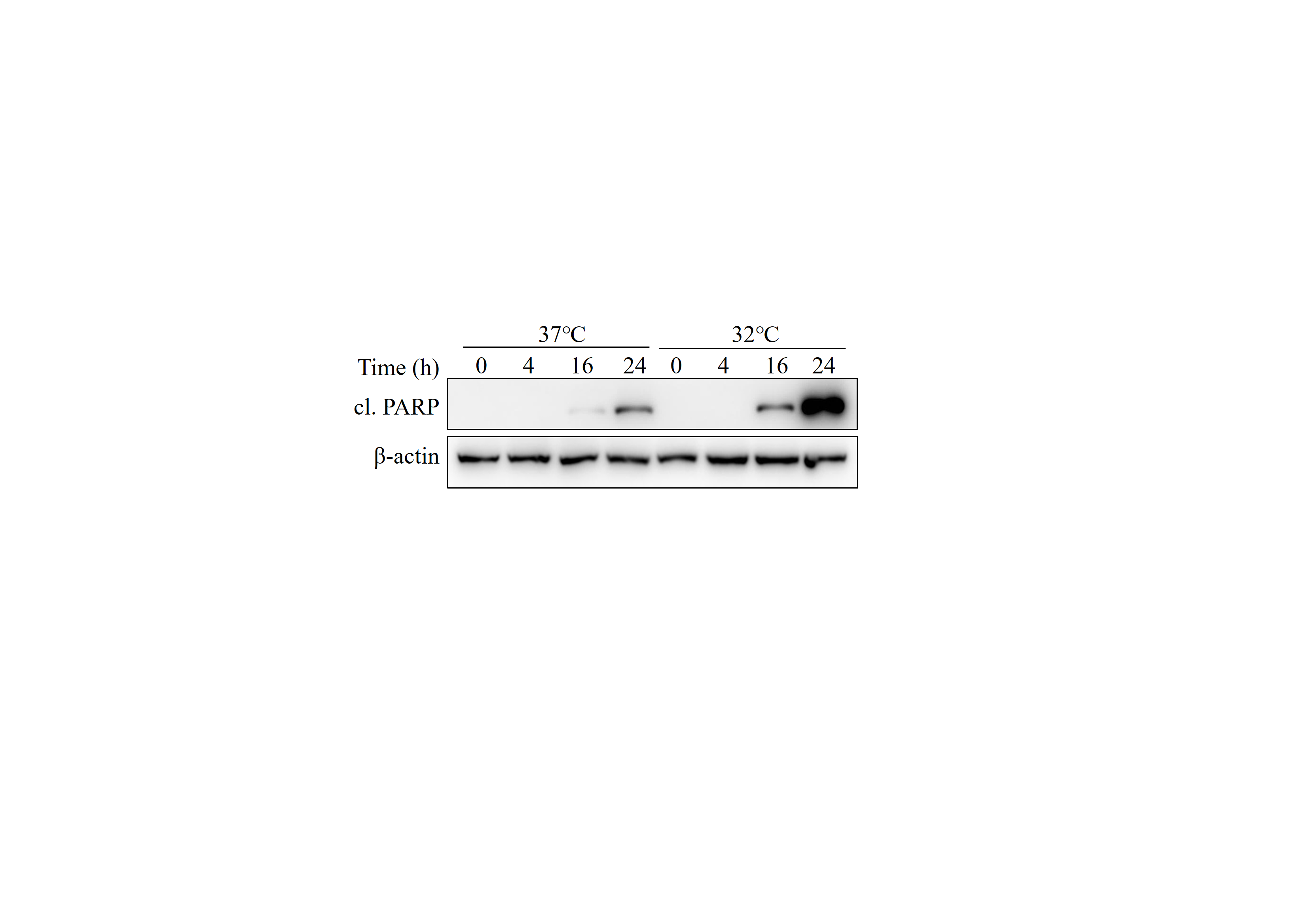

Supplement: Figure S1 — Hypothermia treatment aggravates MPP+-induced apoptosis in SH-SY5Y cells. With the addition of MPP+ (3 mM), cells were incubated at 37 or 32°C for a time course as indicated, and the protein levels of cl. PARP were measured by Western blot. [file Image_1.TIF]

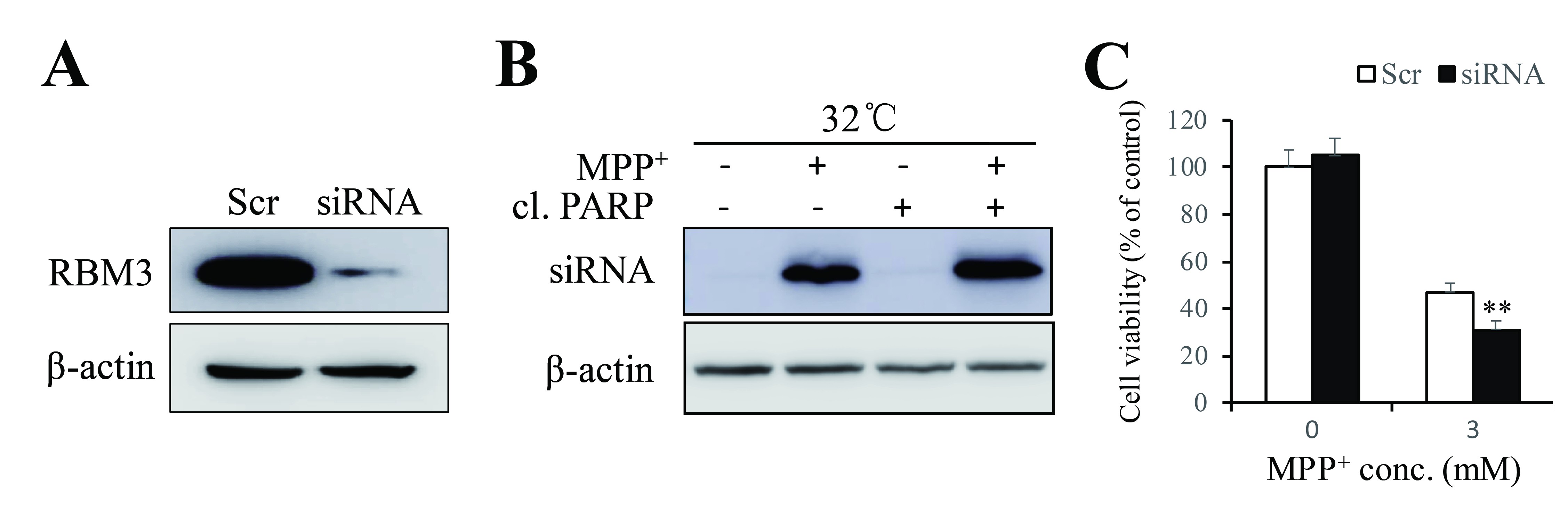

Supplement: Figure S2 — RBM3 silencing aggravates MPP+-induced apoptosis in SH-SY5Y cells. After 2-d transfection of RBM3 siRNA (siRNA) or scrambled siRNA (Src), SH-SY5Y cells were pre-cultured for 1 d at 37 or 32°C and then treated with MPP+ (3 mM) for 1 d. Western blot was performed to detect the protein levels of RBM3 (A) and cl. PARP (B). Under same conditions, cell viability was assessed by MTT assay (C). Multiple comparisons were analyzed by a two-way ANOVA and Bonferroni's post hoc. *Indicates post hoc significant difference compared to control groups (Scr). **P < 0.01. [file Image_2.JPEG]
